# Retraction: Plant rhabdovirus glycoprotein activates unfolded protein response-mediated antiviral ER-phagy in insect vectors

**DOI:** 10.1371/journal.ppat.1014319

**Published:** 2026-06-12

**Authors:** The PLOS Pathogens Editors

After this article [1] was published, co-corresponding author TW contacted the journal to raise concerns that the Fig 1D RSMV- panels are incorrect.

Upon further review of the article, the *PLOS Pathogens* Editors identified additional concerns regarding results presented in Figs 1, 3-5, and S1, and in S1 Data and S1 Raw Images in [1]. Specifically:

- The Fig 1D RSMV- panels in [1] appear similar to the Fig 1D RDV^-^ panels in [2,3].
- The following western blot panels and their respective uncropped blots in the S1 Raw Images file in [1] appear similar despite being used to represent different experimental results:
  - Fig 1H GAPDH, Fig 1N GAPDH, and Fig 3F GAPDH
  - Fig 1N ATF4 and Fig 4H Sec62
  - Fig 1N RSMV N and Fig 3J RSMV N
  - Fig 3H RSMV N and Fig 4H RSMV N
  - Fig 3H GAPDH and Fig S1D Cytoplasm GAPDH
- Fig 5J and Fig 5K appear similar when rotated.
- In S1 Data in [1]:
  - The Fig 3I RSMVG values are the same as the Fig 3K RSMVG values despite being used to represent different experimental results.
  - The Fig 3G RSMV N dsATF4, Fig 3K RSMV N dsPERK, and Fig 3K RSMV G dselF2α results appear to contain more of the same digits than would be expected.

Co-corresponding author TW provided replacement images for the Fig 1D RSMV- panels from the time of the original experiments, including triplicate underlying image data for Fig 1D in [1], and they stated that the Fig 1D RDV^-^ panels in [2] are correct. The *PLOS Pathogens* Editors consider this concern resolved.

In response to the remaining concerns listed above, co-corresponding author TW stated that errors occurred during the preparation of Figs 1, 3, 4, S1, and the S1 Data underlying Fig 3. They provided replacement western blot images for these figures and replacement data for Fig 5J. Co-corresponding author TW requested retraction of the article [1] due to the extent of these errors.

In light of the above concerns that question the reliability and integrity of the reported results, and in line with co-corresponding author TW’s request, the *PLOS Pathogens* Editors retract this article.

All authors agreed with the retraction, stand by the article’s findings, and apologize for the issues with the published article.

In addition to the issues above, the article’s Competing Interests statement is updated to: Author TW was a member of the *PLOS Pathogens* Editorial Board at the time [1] was accepted for publication.
